# Risk factors for disease progression in COVID-19 patients

**DOI:** 10.1186/s12879-020-05144-x

**Published:** 2020-06-23

**Authors:** Min Cheol Chang, Yu-Kyung Park, Bong-Ok Kim, Donghwi Park

**Affiliations:** 1 Department of Rehabilitative Medicine, College of Medicine, Yeungnam University, Daegu, Republic of Korea; 2 Department of Internal Medicine, Korea Worker’s Compensation & Welfare Service Daegu Hospital, Daegu, Republic of Korea; 3 Department of Rehabilitation Medicine, Korea Worker’s Compensation & Welfare Service Daegu Hospital, Daegu, Republic of Korea; 4 Department of Physical Medicine and Rehabilitation, Ulsan University Hospital, University of Ulsan College of Medicine, 877, Bangeojin sunhwando-ro, Ulsan, 44033 Dong-gu Republic of Korea

**Keywords:** Coronavirus disease, Risk factor: symptom aggravation, Chilling, Fever, Diabetes

## Abstract

**Background:**

Coronavirus disease (COVID-19) is rapidly spreading worldwide. Although 10–20% of patients with COVID-19 have severe symptoms, little is known about the risk factors related to the aggravation of COVID-19 symptoms from asymptomatic or mild to severe disease states.

**Methods:**

This retrospective study included 211 patients who were asymptomatic or with mild presentations of COVID-19. We evaluated the differences in demographic and clinical data between the cured (discharged to home) and transferred (aggravated to severe-stage COVID-19) groups.

**Results:**

A multivariate logistic analysis showed that body temperature, chills, initial chest X-ray findings, and the presence of diabetes were significantly associated with predicting the progression to severe stage of COVID-19 (*p* < 0.05). The odds ratio of transfer in patients with COVID-19 increased by 12.7-fold for abnormal findings such as haziness or consolidation in initial chest X-ray, 6.32-fold for initial symptom of chills, and 64.1-fold for diabetes.

**Conclusions:**

Even if patients are asymptomatic or have mild symptoms, clinicians should closely observe patients with COVID-19 presenting with chills, body temperature > 37.5 °C, findings of pneumonia in chest X-ray, or diabetes.

## Background

After the first case of coronavirus disease (COVID-19) in Wuhan, China in late December 2019, the severe acute respiratory syndrome coronavirus 2 (SARS-CoV-2) has spread to over 200 countries in about 3 months. On March 11, 2020, the World Health Organization (WHO) declared the outbreak a pandemic [1–3]. The estimated case-fatality rate for 2019-nCoV is around 3.5%, which is similar to that of Spanish influenza (2–3%) and much higher than that of seasonal influenza (0.1%) [4]. Over 80% of COVID-19 cases have mild symptoms; however, 10–20% of COVID-19 cases proceed to a severe stage [5]. The identification of factors associated with the aggravation of patient symptoms from asymptomatic-mild to severe is essential for providing efficient and appropriate management to patients with COVID-19.

Most studies to date have investigated risk factors of mortality in patients with COVID-19 [6–8]. Older age and history of coronary vascular disease were reported to increase the risk of death from COVID-19 [6–8]. However, little is known about the risk factors related to the aggravation of COVID-19 symptoms from asymptomatic or mild to severe disease states.

The current study recruited patients with COVID-19 who presented no or mild symptoms from a single center in South Korea and evaluated factors aggravating their symptoms to severe-stage disease.

## Methods

### Study design and participants

This retrospective cohort study investigated adult inpatients (≥18 years) from Korea Worker’s Compensation & Welfare Service Daegu Hospital (Daegu, South Korea). We screened all adult patients diagnosed with COVID-19 according to WHO interim guidance and who were discharged by cure or transferred to specialized infectious disease hospitals due to aggravated symptoms between Feb 28, 2020 (i.e., when the first patients were admitted) and March 31, 2020.

The study hospital was originally a public rehabilitation hospital. However, after the large spread of COVID-19 across Daegu city, the government assigned the hospital as a public infectious disease hospital for isolating and managing asymptomatic or mild COVID-19 patients in Daegu. Patients with COVID-19 were classified into four levels (asymptomatic, mild, moderate, and severe) according to the severity of their symptoms (Table 1). The four-classification system was made by Daegu Medical Association for fast classification of patients with COVID-19. Patients with COVID-19 admitted to the study hospital who progressed to severe-stage disease were transferred to specialized infectious disease hospitals with negative pressure rooms. Therefore, we enrolled many patients who were hospitalized for asymptomatic or mild COVID-19. We divided these patients into cured and transferred groups according to their treatment results. The cured group included initially asymptomatic or mildly symptomatic COVID-19 patients who were discharged to home after complete cure. The transferred group included COVID-19 patients with initial asymptomatic or mild stage who were transferred to a specialized infectious disease hospital due to COVID-19 progression to severe stage. This study was approved by the Institutional Review Board of Yeungnam University Hospital and the requirement for informed consent was waived by the Ethics Commission.

**Table 1:** Classification of patients with COVID-19 according to symptom severity

Classification	Classification criteria
Asymptomatic	Asymptomatic or body temperature < 37.5 °C
Mild	Body temperature ≥ 37.5 °C but O_2_ supply not required
Moderate	O_2_ supply via nasal or venturi mask required
Severe	High-flow O_2_ supply or mechanical ventilation required

### COVID-19 treatments

All hospitalized patients were administered antibiotics and antiviral treatment. The antibiotics included azithromycin 500 mg (Aziromax®) and cefixime 200 mg (Pocef®) peroral. The antiviral treatments were either lopinavir/ritonavir 200 mg/50 mg (Kaletra®) two tablets per day alone or a combination of lopinavir/ritonavir 200 mg/50 mg (Kaletra®) two tablets and hydroxychloroquine sulfate 400 mg (Oxiklorine®) peroral.

### Data collection

We extracted clinical, epidemiological, demographic, medication, and outcome data from patient electronic medical records. Two physicians (M.C and D.P) independently investigated all data using a standardized data collection form.

### Laboratory procedures

The diagnosis of COVID-19 was performed using a real-time polymerase chain reaction (RT-PCR) kit approved by the Korean Centers for Disease Control and Prevention (KCDC) and Korean Ministry of Food and Drug Safety [9].

### Risk factors

We investigated the chronic medical underlying diseases of the patients, including diabetes, chronic kidney disease (CKD), chronic liver disease, chronic lung disease, chronic cardiovascular disease, carcinoma, dyslipidemia, and hypertension, according to the National Health Insurance System of South Korea diagnosis codes and based on the results of previous studies on the risk factors for COVID-19 and classification system announced by the KCDC. Chronic lung disease was defined as chronic obstructive pulmonary disease (COPD), asthma, interstitial lung disease (ILD), idiopathic pulmonary fibrosis (IPF), or bronchiectasis. We also investigated the presence of allergic rhinitis in patients with COVID-19 and assessed the initial symptoms and vital signs, such as cough, sputum, myalgia, chills, rhinorrhea, dyspnea, chest pain, body temperature, and oxygen saturation in pulse oximetry.

### Statistical analysis

We performed independent t- and chi-square tests to assess differences in demographic and clinical data between the cured and transferred groups. Moreover, we also used multivariate logistic analysis through the forward selection method to analyze the correlations between clinical parameters (underlying diseases, initial symptoms, and initial vital signs) and progression to severe disease stage in patients with COVID-19. Finally, we performed a receiver operating characteristic (ROC) analysis in each group to evaluate the accuracy of predictive factors for body temperature for the progression to moderate-stage disease in patients with COVID-19. All statistical analyses were conducted using IBM SPSS Statistics for Windows, version. 22.0 (IBM Corp., Armonk, NY, USA).

## Results

### Demographics and clinical characteristics of patients with COVID-19

The final analysis in this study included 211 adult patients hospitalized at Korea Worker’s Compensation & Welfare Service Daegu Hospital with COVID-19 before March 31, 2020. Thirteen patients transferred to a specialized infectious disease hospital with negative pressure rooms during hospitalization and 198 patients were discharged to home. The median age of the 211 patients was 37.55 ± 14.53 years (range: 18 to 74 years) and the sex ratio was 74:137 (male:female). Comorbidities were present in 33 patients (15.6%), with hypertension (13 patients, 6.2%) the most common, followed by allergic rhinitis (nine patients, 4.3%) and dyslipidemia (six patients, 2.8%) (Table 2).

**Table 2:** Clinical characteristics of patients with COVID-19 on admission

Variable	Total	Cured Group	Transferred Group	*P*-value
Total, *n* (%)	211 (100%)	198 (93.8%)	13 (6.2%)	
Age, years	37.55 ± 14.53	36.47 ± 14.04	54.08 ± 11.98	.000*
Sex, *n* (%)				
Female	137 (64.9%)	129 (65.2%)	8 (61.5%)	.791
Male	74 (35.1%)	69 (34.8%)	5 (38.5%)	
Initial vital sign & symptoms				
Body temperature (°C)	37.09 ± 4.85	37.04 ± 0.42	37.81 ± 0.76	.003*
Oxygen saturation (%)	97.20 ± 11.88	97.24 ± 11.93	96.57 ± 9.76	.096
Chills	22 (10.4%)	16 (8.1%)	6 (46.2%)	.000*
Cough	92 (43.6%)	83 (41.9%)	9 (69.2%)	.054
Myalgia	44 (20.9%)	35 (17.7%)	9 (69.2%)	.000*
Sputum	66 (31.3%)	59 (29.8%)	7 (53.8%)	.070
Rhinorrhea	58 (27.5%)	52 (26.3%)	6 (46.2%)	.120
Sore throat	46 (21.8%)	43 (21.7%)	3 (23.1%)	.908
Headache	53 (25.1%)	47 (23.7%)	6 (46.2%)	.071
Diarrhea	33 (15.6%)	30 ((1.5%)	3 (23.1%)	.446
Dyspnea	26 (12.3%)	21 (10.6%)	5 (38.5%)	.003*
Chest pain	24 (11.4%)	22 (11.1%)	2 (15.4%)	.643
Findings of pneumonia in chest X-ray	54 (25.6%)	43 (21.7%)	11 (84.6%)	.000*
Comorbidities				
Hypertension	13 (6.2%)	9 (4.5%)	4 (30.8%)	.000*
Diabetes	4 (1.9%)	1 (0.5%)	3 (23.1%)	.000*
Chronic kidney disease	0 (0%)	0 (0%)	0 (0%)	.999
Dyslipidemia	6 (2.8%)	4 (2.0%)	2 (15.4%)	.005*
Chronic lung disease	6 (2.8%)	6 (3%)	0 (0%)	.524
Allergic rhinitis	9 (4.3%)	9 (4.5%)	0 (0%)	.432
Carcinoma	1 (0.5%)	1 (0.5%)	0 (0%)	.797
Cardiovascular disease	2 (0.9%)	2 (1.0%)	0 (0%)	.716

*p*-values were calculated by independent T- or chi-square tests, as appropriate. *Significant difference between groups (*p* < 0.05). COVID-19: coronavirus disease 2019. Values: mean ± standard deviation

### Risk factors for progression to moderate-stage COVID-19

Comparison of demographic and clinical data between the cured (discharged to home) and transferred (to specialized infectious disease hospital) groups showed a significant difference in age (*p*-value < 0.05) (Table 2). Comparison of the initial vital signs and symptoms between the cured and transferred groups showed significant differences in body temperature, initial chest x-ray findings, chills, myalgia, and dyspnea (*p* < 0.05) (Table 2).

Comparison of the underlying comorbidities between the cured and transferred groups showed significantly higher presence of hypertension, diabetes, and dyslipidemia in the transferred group than in the cured group (*p* < 0.05) (Table 2). The presence of cancer, chronic lung disease, cardiovascular disease, and allergic rhinitis did not differ significantly between the cured and transferred groups.

Multivariate logistic analysis showed that body temperature, chills, initial chest x-ray findings, and the presence of diabetes were significant parameters predicting the progression to severe-stage COVID-19 (*p* < 0.05) (Table 3). The odd ratios of transferred patients with COVID-19 increased by 12.7-fold for abnormal findings such as haziness or consolidation in initial chest x-ray, 6.32-fold for initial chills, and 64.1-fold for diabetes (Table 4).

**Table 3:** Risk factors associated with progression to severe-stage COVID-19

Parameter	Beta coefficient	Standard error	Multivariable OR (95% CI)	*p*-value
Age	.060	.046	1.061 (0.971–1.161)	.192
Body temperature	.216	.070	1.241 (1.082–1.424)	.002*
Dyspnea	2.049	1.454	7.760 (0.6449–134.131)	.159
Myalgia	1.654	1.082	5.227 (0.627–46.550)	.126
Chills	1.844	.855	6.321 (1.183–33.772)	.031*
Findings of pneumonia in chest x-ray	2.541	.98.	12.690 (1.86.–86.602)	.010*
Diabetes	4.161	1.345	64.134 (4.593–895.479)	.002*
Hypertension	1.091	1.866	2.976 (0.770–115.224)	.559
Dyslipidemia	.185	2.459	1.203 (0.010–148.987)	.940

*p*-values were obtained by multivariate logistic analysis *Significant difference noted (*p* < .05)

*OR* odds ratio, *CI* confidence interval

**Table 4:** ROC curve analysis of body temperature for progression to severe-stage COVID-19

Parameter	AUC	Standard error	*p*-value	95% CI
Body temperature	.742	.052	.000^*^	0.646–0.936

*Significant difference noted (*p* < .05)

*ROC* receiver operating characteristic, *AUC* area under the ROC curve, *CI* confidence interval

**Fig. 1:**
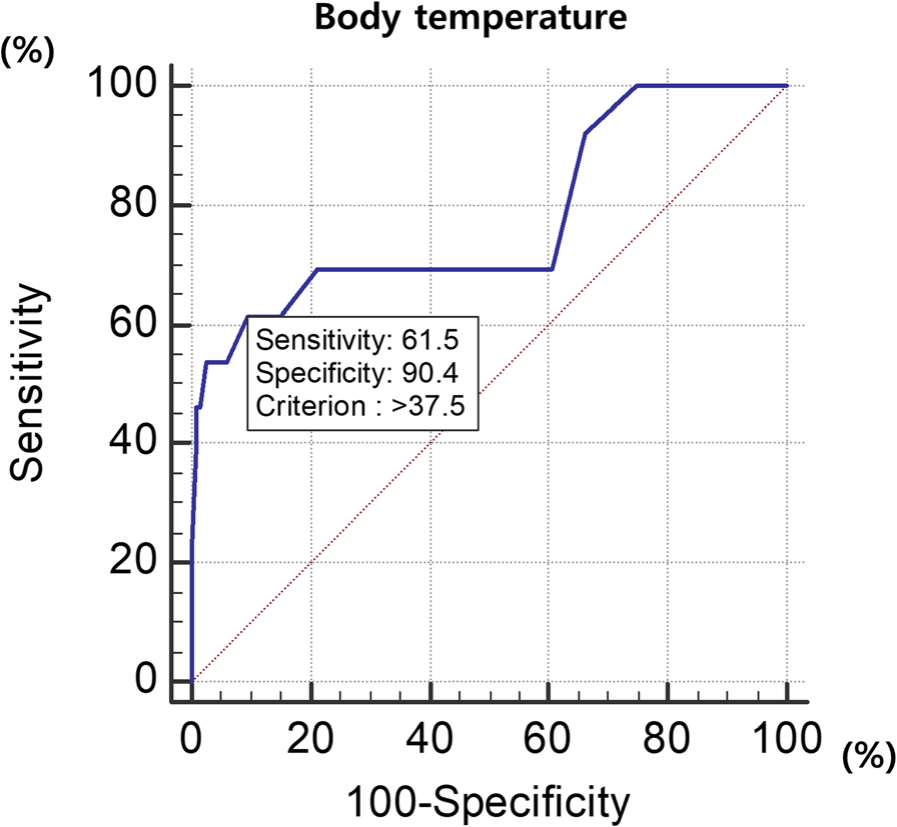
The area under the receiver operating characteristic (ROC) curve for age predicting non-survival was 0.742 (95% confidence interval [CI], 0.646–0.936; *p* < 0.001). The optimal cutoff value obtained from the maximum Youden index J was 37.5 (sensitivity: 61.5%, specificity: 90.4%) and the odds ratio of progression to severe COVID-19 increased by 1.24-fold for every 0.1 °C increase in body temperature

In patients with COVID-19, the area under the ROC curve of body temperature for predicting progression to severe-stage COVID-19 was 0.742 (95% CI, 0.646–0.936; *p* < 0.001) (Table 4). The optimal cutoff value obtained from the maximum Youden index J was 37.5 °C (sensitivity: 61.5%, specificity: 90.4%) and the odds ratio of progression to severe-stage COVID-19 increased by 1.24-fold for every 0.1 °C increase in body temperature (Fig. 1).

## Discussion

In this study, we found that chills, body temperature > 37.5 °C, abnormal findings such as haziness or consolidation on initial chest X-ray, or diabetes were risk factors for aggravation of COVID-19 symptoms from asymptomatic-mild to severe.

Among patients with diabetes, the odds ratio for progression to severe-stage COVID-19 was about 60 times higher than that of COVID-19 patients without diabetes, which was the highest ratio among statistically significant aggravating factors in our study. The high risk of progression to severe-stage COVID-19 in patients with diabetes is likely because of hyperglycemic conditions that cause immune dysfunction including impaired neutrophil function, antioxidant system function, and humoral immunity [10–12]. Additionally, patients with diabetes are vulnerable to nosocomial infection, which can deteriorate their general condition and aggravate COVID-19 symptoms [13].

Other than diabetes, chills, body temperature > 37.5 °C, and abnormal findings on initial chest X-ray such as haziness or consolidation were risk factors for proceeding to severe-stage disease. Chills and fever are responses to released inflammatory mediators such as cytokines and chemokines [14, 15]. These inflammatory mediators cause tissue damage and organ dysfunction by stimulating toxic oxygen derivatives [16–18]. Accordingly, chills and fever can be clinical signs indicating poor patient prognosis. Also, lung haziness or consolidation on chest X-ray indicated the significant effects of 2019-nCoV, suggesting the high possibility of progression to severe conditions in patients with these abnormal findings.

Previous studies reported age to be the most important predictor of death in patients with COVID-19 [6, 8]. In contrast, in our study, age was not a predictive factor for symptom aggravation in these patients This inconsistency may be because the patients enrolled in our study were relatively young (average age: 37.6 years). Also, our primary outcome was not risk factors for mortality but rather factors for symptom aggravation. However, although the difference was not statistically significant, we observed a tendency for older patients (average age: 54.1 years) to progress to severe-stage disease compared to relatively younger patients (average age: 36.5 years). The age-dependent functional defects in immunologic cells lead to impaired suppression of viral replication [19–21].

In 2020, some studies evaluating the risk factors of mortality in patients with COVID-19 reported higher death rates with increasing age [8, 11]. Regarding underlying diseases, among 416 hospitalized patients with COVID-19 in Wuhan, China, [7, 6] Shi et al. observed a higher mortality rate in 82 patients with cardiac injury rate than that in 334 patients without cardiac injury [7]. However, to our knowledge, no other study has yet evaluated factors associated with the aggravation of COVID-19 symptoms. Therefore, our study is first to report factors aggravating COVID-19 symptoms.

## Conclusion

In conclusion, we found that asymptomatic patients or patients with mild symptoms including chills, body temperature > 37.5 °C, findings of pneumonia on chest X-ray, or diabetes mellitus were prone to developing severe-stage COVID-19. Therefore, clinicians should consider these potential risk factors for symptom aggravation during management of COVID-19 patients. Our study has some limitations, including the relatively small number of patients, particularly those with increased symptom deterioration. Also, we recruited only patients with asymptomatic-mild-stage and not moderate or severe-stage disease. Therefore, further studies with larger patient numbers and patients with various stages of disease are warranted.

## Data Availability

The datasets used and/or analysed during the current study are available from the corresponding author on reasonable request.

## Abbreviations

COVID-19: Coronavirus disease
CKD: Chronic kidney disease
COPD: Chronic obstructive pulmonary disease
ILD: Interstitial lung disease
IPF: Idiopathic pulmonary fibrosis
ROC: Receiver operating characteristic
KCDC: Korean Centers for Disease Control and Prevention
WHO: World Health Organization
